# Does organizational vision really matter? An empirical examination of factors related to organizational vision integration among hospital employees

**DOI:** 10.1186/s12913-021-06503-3

**Published:** 2021-05-21

**Authors:** Terje Sltten, Barbara Rebecca Mutonyi, Gudbrand Lien

**Affiliations:** Inland Norway University of Applied Sciences, Campus Lillehammer, 2604 Lillehammer, Norway

**Keywords:** Organizational vision integration, Creative performance, Organizational attractiveness, Psychological capital, Employees, Hospital

## Abstract

**Background:**

There seems to be a consensus that a vision for an organization is a valuable thing for organizations to have. However, research on organizational vision has predominantly been studied from a leadership perspective. In contrast to previous research, organizational vision in this paper takes an employee perspective. Specifically, the purpose is to examine factors associated with the integration of organizational vision among employees in hospital organizations. Consequently, it focuses on a relatively neglected domain within health services research.

**Methods:**

A conceptual model, centred on the concept of organizational vision integration, was developed and tested on a sample (*N*=1008) consisting of hospital employees. Partial least-squares structural equation modelling (PLS-SEM) was used to test the hypotheses, using SmartPLS 3 software. Furthermore, a bootstrapping test was used to inspect potential mediating effects. Specifically, the test assessed whether the proposed direct and indirect effects were statistically significant, and at the same time revealed the nature of the mediation effect.

**Results:**

The results from the empirical study reveal three key findings: i) organizational vision integration among employees is directly and positively related to creative performance in their respective work role (** = 0.16). Organizational vision integration and employees psychological capital explains almost 40% (*R*^2^ = 0.36) in employees creative performance, ii) psychological capital and employees perception of organizational attractiveness are directly and positively related to employees organizational vision integration (** = 0.19 and ** = 0.40, respectively) and explains about 30% (*R*^2^ = 0.29) of employees organizational vision integration, iii) employees organizational vision integration mediates the relationship between employees psychological capital, perception of organizational attractiveness and employees creative performance.

**Conclusions:**

Taking an employee perspective, this study contributes to revealing whether and how organizational vision matters and its impact on hospital employees work performance. To achieve organizational vision integration among hospital employees successfully, this study shows that it is important for hospital leaders to be aware of the pattern of impact of both personal as well as environmental-related factors.

**Supplementary Information:**

The online version contains supplementary material available at 10.1186/s12913-021-06503-3.

## Background

Almost all of todays organizations have developed a strategic vision for their organization. Hospital organizations are no exception to this trend. For example, the organizational vision of the Mayo Clinic in America, which is one of the most famous and best hospitals in the world, is: *Mayo Clinic will provide an unparalleled experience as the most trusted partner for health care* [1]. Another example is the Karolinska Institutet in Sweden, which is one of the worlds foremost medical universities. The organizational vision of Karolinska Institutet is: *to advance knowledge about life and strive towards better health for all* [2]. There seems to be, both in practice and theory, a consensus and an agreement that a vision is a good and valuable aspect for organizations to have. The fundamental premises or basic idea for such an assumption is the belief that organizational vision works just like a lodestar for a ship that is used to guide a ship (in our case hospital organizations) towards its desired direction and preferred aim. However, on the other hand, some people question or wonder whether a vision of an organization really has an impact, expressing statements such as: after all, a vision of an organization is just some few words written on a piece of paper or on the wall of a company. According to Liu, only a few companies produce vision statements that actually capture the hearts of the group members [3]. In a similar vein, Kantabutra and Avery timely ask: some organizations already have a vision, but how effective is it? [4]. This signals a need to acquire more insight and knowledge about factors associated with organizational vision.

In previous research, one finds a variety of descriptions of the concept of organizational vision. However, most descriptions of organizational visions have a dominating leadership perspective in its orientation towards goal achievement. This dominating leadership focus can be criticized because it does not capture well or correspond satisfactorily to three aspects of how to integrate an organizational vision in todays modern organizational business context. Regarding this, Kohles et al. state: as organizations become increasingly horizontal simply crafting a compelling vision may no longer be enough [5]. This horizontal approach implies that leaders should be and act more like employee supporters than employee supervisors [6]. Leaders undoubtedly play an important and active role in developing a vision integration. However, it is not enough to just communicate or spread the vision throughout the organization. Kohles et al. stated that such approaches assume that leaders need only to articulate a vision to achieve follower buy-in [5]. This stresses the importance to also consider the integration of organizational vision from an organizational members perspective. These receivers of the proposed organization vision play a critical role because they ultimately determine whether vision statements are ignored or accepted [5]. Although leaders follow all how-to-do-it recipes to ensure that employees accept the organizational vision, it is not sufficient. Employees mental acceptance of an organizational vision does not necessarily mean it will guide them in how to perform their work. The acceptance should also include a behavioural manifestation of organizational vision in employees day-to-day work. Accordingly, organizational vision should be embedded in employees work role performance. Stated in another way: organizational vision should be integrated into the minds and feet of organizational members. Therefore, an organizational vision integration can only be considered as successfully achieved when it provides conscious directions and acts as an inner mental voice that guides the behaviour of organizational actors [5]. Consequently, the following can be concluded: leaders can create and persuasively sell the most brilliant organizational vision in the world, but it takes employees to bring the organizational vision to (real) life and reality. Accordingly, it becomes fundamentally important to take an employee perspective when studying the integration of organizational vision.

For the reasons above, this paper has three aims, all with their own associated contributions. First, and most importantly, it aims to take an employee perspective when studying organizational vision integration. As such, it contributes to the focus on group organizational members (referring to employees) that have been only rarely mentioned in the visioning process often relegated to a largely passive role in vision implementation [7]. Second, it aims to examine how organizational vision integration is associated with the achievement of desired organizational goals. Specifically, it focuses on whether an organizational vision can increase hospital employees level of creativity and innovative behaviour (referred to as creative performance) in such a way that it is beneficial to the performance of hospital employees work role. As such, it responds to the call for more research on conditions that promote the innovative performance [which is an area of research] that still remain to be explored more in depth [8]. Recently, Mutonyi et al. called for more research that links organizational vision to employees capability to be innovative [9]. Third, it aims to examine a selection of premises or factors that potentially trigger the integration of organizational vision among employees as well as employees work role performance. Specifically, it includes both personal-related factors (referred to as employees psychological capital) as well as environmental-related factors (referred to as organizational attractiveness). By conceptually suggesting and empirical testing organizational vision as a mediating factor, the paper add to our knowledge regarding the role organizational vision plays for hospital organizations.

To the authors best knowledge, this is one of the pioneering studies within health services research that has focused on aspects associated with hospital employees integration of organizational vision. According to Liu [3], there is a limited collection of materials relative to organizational vision particularly those applicable to the service industrys critical area of service quality [3]. Consequently, the paper contributes to both theory and practice, regarding organizational vision, within the domain of health services research.

The paper is organized into three parts. The first part elaborate the conceptual framework of the study. The second part contains the methodology, statistical analysis and results from the tests of hypotheses. The final part discusses the findings from the study and provide suggestions for further research as well as a final conclusion of the study.

### Conceptual framework of the study

#### Organizational vision integration (OVI)

Organizational vision is described in various and multifaceted ways in the literature. To name a few examples, organizational vision is described in terms of being an ideal and unique image of the future [10], a mental image of a possible and desirable future state of the organization [11], a business technology, or corporate culture in terms of what it should become over the long term and articulate a feasible way of achieving this goal [12], a guide to what the organization should become rather than a description of what it is [13], ideological goal that organization members can feel morally satisfied in pursuing [14], the primary guiding force of all organizational activity [15]. Others suggest that an organizational vision should be motivational, build self-confidence and create a common purpose among those who are encompassed by the vision [16].

Previous research within the domain of organizational vision has, according to Testa [17], been dominated by three streams of research. First, research has taken a leader level and focused on vision as characteristics or traits of effective leaders. Second, research has focused on how vision is defined and the development of vision statements. Third, research has focused on the role vision plays in the achievement of organizational goals as a by-product of leadership style [17]. A predominantly common trait across the three research streams is, as noted above, to take a leadership perspective. Consequently, an employee perspective when studying aspects related to organizational vision has to a large extent been neglected. Therefore, instead of considering a top-down approach, regarding such as how leaders communicate or diffuse the organizational vision through the organization, the approach of this study embraces how well the organizational vision actually is adopted, absorbed or integrated among individual members of the organization.

Employees focus and role in the process of OVI are only rarely or tangentially discussed as passive recipients of the vision [5]. However, in contrast to this dominating leadership perspective in previous research, this study takes an employee perspective when studying the integration of organizational vision. By taking an employee perspective, it supports Kohles et al.s assumption that the realizations of vision [seen from an employee perspective] may be at least equal to, if not greater than, the importance of strategic leaders [5]. Specifically, in line with Kohles et al., OVI in this study refers to whether or not followers [referring to employees] use the vision as a guiding framework when making decisions and discretionary behaviors in their daily work roles [7]. Consequently, the focus in this definition is on the implementation of the vision in employees minds and feet. The authors of this paper are not aware of any previous study within health services research that has taken an employee perspective when studying hospital employees OVI.

In understanding OVI, one must consider two conditions. First, there is the attention and knowledge. This is about employees perception of whether they have been informed and know and understand the vision [7]. The second condition of OVI, is intention and embraces whether employees use the vision as a guiding framework in their particular jobs [7]. Only when the two conditions are present simultaneously and intertwined do they characterize and constitute a positive OVI. The way that OVI is defined and operationalized in this study is conceptually close to and matches well the concept of Sltten and Mehmetoglu where they refer to strategic attention [18]. Similar to the concept of OVI, Sltten and Mehmetoglu describe strategic attention as how the firms strategy serves as a guiding principle or a compass for frontline employees in their work role [18]. Although the authors label their concepts as strategic attention, it is interesting to note that the items used for their concept focus exclusively on aspects related to the integration of organizational vision and thus overlap to a large extent how the concept of OVI is described and operationalized in this study. Considering the important role employees play in all so-called service organizations, such as hospital organizations (which is the empirical context of this study), it is reasonable to assume that it is of fundamental importance to integrate or implement organizational vision into each individual member of the organization. As Sltten and Mehmetoglu noted: Without implementation the organizations strategy is useless implementation is fundamental for a firms success [18].

In the following sections, several factors are proposed to be related, either indirectly or directly, to employees OVI.

#### The relationship between OVI, CP and PsyCap

**Fig. 1:**
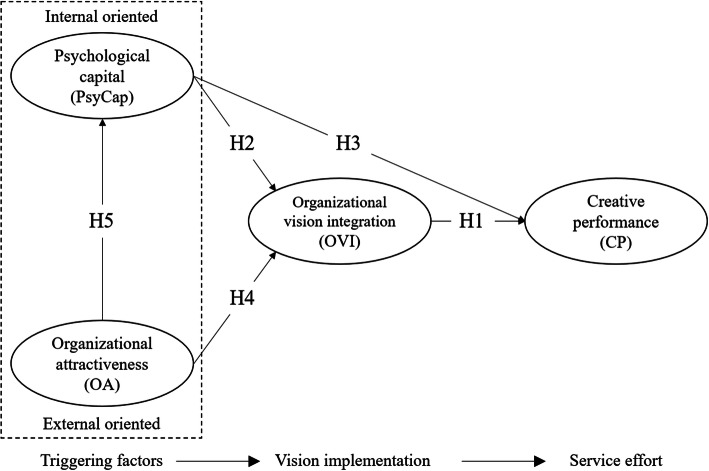
Conceptual model of examining factors related to organizational vision integration

Employees OVI is suggested to be related to employees *creative performance* (CP). CP is a service effort which is manifested in employees respective work roles. CP in this study refers to and embraces individual employees capability to be creative and innovative. Consequently, CP is a combination of both a cognitive element (think creatively) and a behavioural element (act innovatively). In the literature, one will find that creativity and innovative behaviour are two closely related concepts. For example, Gilmartin suggests that creativity is the fuel for innovation and asserts that creativity is a basic building block of invention and thus innovation [19]. The two elements that constitute CP are manifested in employees respective work role in the organization. It is important to note that CP is not limited or directed towards any specific work role. In contrast, CP as one part of what in Fig.1 is labelled as employees service effort could be manifested both internally in the organization (e.g. a new administrative routine) or externally (e.g. a new way that improves service quality provided to hospital patients). Consequently, CP could be potentially manifested in various work roles employees hold in the organization.

As also indicated in the previous discussion, an organizational vision is a tangible representation of the long-term goals for the company and its idealized future state [5]. This means that a vision of an organization per definition communicates to organizational members the existence of a gap between the current situation and the ideal future state of an organization. The only way to close the gap is by undertaking changes that move the organization towards its desired direction and preferred aim. Consequently, when an organizational vision is appropriately designed it constitutes a form for freedom, encouragement and motivation for organizational members to make the necessary behavioural changes to become more in harmony and in accordance with the organizations desired and idealized future state. This is in line with Kohles et al. who state: while vision statements may be more or less novel, ranging from incremental shifts to drastic changes, all of them represent an attempt to change employee behaviors [7]. Consequently, OVI should enable employees to think creatively and be innovative and thus act visionary within the boundaries of the formal vision articulated by the organization. In the presence of OVI, employees have both knowledge about organizational vision and consciously use the organizational vision as a guiding tool when performing their work role. Little research has attempted to explore the relationship between OVI and CP in health services research. For example, in a study by Sltten and Mehmetoglu [18], which included 279 employees in hospitality organizations, the authors found that employees strategic attention (a concept similar to OVI) was positively associated with employees level of work engagement and level of innovative behaviour. Consequently, when OVI is present it is reasonable to assume that such employees have a larger potential to manifest a greater CP than those with less OVI. Consequently, the following first hypothesis is proposed:

- **Hypothesis 1:** *OVI is positively related to CP*

The level of OVI is also suggested to be related employees *psychological capital* (PsyCap). PsyCap is an internal oriented triggering factor. The expression internal oriented reflects that PsyCap is an individual or personal-related factor. The PsyCap of a person can be explained as the positive psychological state of the individual towards positive development [20]. PsyCap consists of four personal-related resources, which are (i) hope, (ii) efficacy, (iii) resilience and (iv) optimism [20]. These four resources of a person have a synergistic association and collectively constitute a state-like resource of who you are [21]. In line with previous research, this study defines PsyCap as an individuals positive psychological state characterized by: (1) having confidence (self-efficacy) to take on challenging tasks and put in the necessary effort to succeed at them; (2) having a positive feeling (optimism) about future success; (3) persevering towards goals, and when necessary redirecting paths to goals (hope) to succeed; and (4) when beset by problems and adversity, bouncing back, sustaining or increasing ones efforts (resilience) to attain success [20].

The concept of PsyCap is about the level of positive resources or internal strengths a person possesses that can be capitalized on or exploited. Thus, the resources of PsyCap constitute a persons motivational state. As Abbas and Raja state, psychological resources have motivational components [22]. Thus, regarding the role employees possess in the organizations, PsyCap should make employees more engaged, more open for changes and more eager to get things done as prescribed by the organization (e.g. through the organizational vision). Previous research supports that PsyCap is positively linked to an increased level of employees work engagement as well as their job performance [23]. Based on this, there are good reasons to expect that the resources of employees PsyCap are also capable of contributing positively to increase OVI of members of an organization. As previously discussed, OVI contains two conditions, referring to (i) attention and (ii) intention. This implies that to achieve OVI there must be an inherent willingness in place among employees, finally determining whether vision statements are ignored or accepted [5].

Therefore, successfully to achieve OVI, employees must have an inner-motivational drive that pushes them to undertake changes in the direction of a continuously improved goal-fulfilment of an idealized future articulated through the vision of the company. A persons level of PsyCap constitutes this necessary motivational driver to OVI. As Abbas and Raja state: PsyCap is considered an individual-level higher-order factor that facilitates change [22]. Considering the four resources embraced in PsyCap (that is hope, efficacy, resilience and optimism [20]) it is reasonable to assume that each individual resource, as well as collectively, has a potential to contribute positively to the successful achievement of OVI. Therefore, the PsyCap of employees can be said to be a prerequisite for the achievement of OVI among organizational members. Consequently, depending on the level and content of employees PsyCap, it should either promote or inhibit the OVI among organizational members. In this study, we limit our focus to examining the positive aspects of PsyCap. Specifically, it is expected that the more PsyCap possessed by employees the more it positively relates to the OVI among organizational members. Consequently, the following hypothesis is proposed:

- **Hypothesis 2:** *PsyCap is positively related to OVI*

Although PsyCap is expected to have impact on OVI the literature also suggest that PsyCap are directly related to CP. For example, Abbas and Raja state that based on the four resources that constitute PsyCap: these positive psychological resource capacities may help employees exhibit innovative behaviors [22]. Previous research has identified a positive direct relationship between PsyCap, both as an individual and a collective resource, and CP in a variety of employee contexts [22,27]. However, very little research has been undertaken examining the relationship between PsyCap and CP using hospital employees as the empirical setting. Based on previous research, this study proposes PsyCap to have a direct effect on employees CP. Consequently, it is assumed that PsyCap will provide a necessary repository of psychological resources that help effectively innovative work-related ideas [22]. Consequently, the following hypothesis is proposed:

- **Hypothesis 3:** *PsyCap is positively related to CP*

#### The relationship between OVI, OA and PsyCap

In this study, employees perception of *organizational attractiveness* (OA) is proposed to be related to employees OVI. OA is an external oriented triggering factor. The expression external oriented reflects that OA embraces an individual employee evaluation and perception of the environment in the organization, whether it is considered as good or bad. Thus, OA in this study is defined as employees perception and the degree employees experience the organization as a great place to work and consider their organization as an attractive place of employment. Specifically, the concept of OA is about peoples attitude toward the organization for which they work [28]. In line with previous definitions of OA [28, 29] the content of OA reflects two aspects of employees attitude, namely, (i) choosing the same organization or employer again if given the choice and (ii) recommending the organization or employer to someone you know well [28]. This definition captures both the direction of the attitude (positive or negative) as well as the strength of the attitude of current employees in the organization [28]. Notably, the definition of OA does not focus on any specific or attribute-like aspects of the organization that employees would potentially find attractive (e.g. leadership support, co-operation etc). In contrast, the definition of OA takes a holistic perspective of what is included in the OA equation. Consequently, OA is an expression of employees attitude that embraces all aspects of the organization that employees find relevant to appraisal. The concept of OA is relatively similar to psychological climate in that it is about individuals interpretation of the environment [e.g. organization] in a way that is psychologically meaningful [30]. Thus, OA reflects employees attitude toward viewing the organization as a desirable entity with which to initiate some relationship [31]. As Sltten et al. commented regarding the definition of OA chosen in this study: it is reasonable to assume these two aspects [of OA] capture well the core objective for any company to strive toward [28].

There are good reasons to expect that OA is related to OVI. When employees perceive OA as favourable, it should lead to a positive attachment to the organization. Based on this, one should expect such employees to be engaged, willing to work harder and thus more proactive to do what is in the interest of the organization that employs them while the opposite would most likely be the case for those who have a negative perception of OA. Previous research on OA, undertaken in a hospital setting, has found that OA is positively related to employees engagement and level of service quality. OA is also found to decrease employees turnover intentions significantly [28]. Consequently, a positive perception of OA implies that employees have a strong identification with their organization. As Chen et al. noted organizational identification reflects the general satisfaction of employees with their organization and their assessment of attractiveness [32]. These employees are likely to continue working for the organization and make their best effort to benefit the organization [32]. Following this, it is expected that those employees who perceive OA as positive are also more inclined to be more motivated to expend necessary effort regarding OVI in their respective work role. A study by Kirkpatrick and Locke supports this idea. In their laboratory study, the authors documented a positive relationship between the attitude of employees and vision implementation [33]. Similarly, in a study by Liu, which included 600 employees, the authors found a positive relationship between employees perception of organizational vision and employees job satisfaction [3]. The concept of satisfaction in the study by Liu captured employees perception of company factors and supervisor factors [3] and thus shares some similarities with the concept of OA. The assumption of a link between OA and OVI can also be drawn from the psychological-contract theory which is one of the most influential theories to understand organizational behavior [29]. Psychological-contract theory focuses on how working relationship is interpreted, understood and enacted [29]. Sltten et al. commented on the relevance of psychological-contract theory for OA: it is reasonable to assume that OA implicitly includes a psychological-contract element that potentially binds the employee to his or her organization [28]. A consequence of this positive binding is, according to Lee et al., that employees psychological contracts influence their efforts on behalf of the employer [34]. When employees perceive their working relationship in the organization as favourable (e.g. perceive OA in a positive way), this has a positive impact on employees effort and motivation to engage more actively in what constitutes an extra-role effort of workers. As such, the OVI of employees stems from a voluntary will do (or psychological contract), and thus not part of a formal written have to do contract (or employment contract). Therefore, it is reasonable to assume that OA acts as an external oriented motivational triggering factor (as presented in Fig. 1) to the OVI of employees. Consequently, the following hypothesis is proposed:

- **Hypothesis 4:** *OA is positively related to OVI*

As previously mentioned, this paper defines PsyCap as a positive psychological state of the individual towards positive development [20]. When defining the concept as a psychological state, it implies that PsyCap is dynamic and prone to change. Specifically, it means that all four resources embraced in PsyCap (referring to (i) hope, (ii) efficacy, (iii) resilience and (iv) optimism [20]) are all potentially changing as time passes. An implication of this is that PsyCap is open for development and therefore is manageable. Luthans et al. support this assumption by stating that PsyCap is open to development and can be managed for effective work performance [21]. By this line of reasoning, it is expected that OA has the potential to positively develop or manage employees PsyCap. To the authors knowledge, no previous research has examined this specific relationship. However, OA is an employees interpretation of the environment [30] and is reflected in their attitude, which embraces all aspects of the organization that employees find relevant to appraisal. When employees have a positive perception of OA, it implicitly means they experience a state of well-being and/or thriving, both of which have been found to be related to PsyCap in previous studies. Therefore, one should expect OA to have similar direction and association with PsyCap as other positive and attractive evaluated aspects of the organizational environment manifested in previous studies. Previous research has found that employees perception of an organizations supportive climate, such as authentic leadership, can positively develop employees PsyCap [23]. In a study by Choi [35], the author found a positive association between employees perception of an organizations autonomous work environment and employees PsyCap. The same positive pattern of relationships, as found in previous studies, is expected to be identified between employees perception of OA and their PsyCap. Consequently, the following hypothesis is proposed:

- **Hypothesis 5:** *OA is positively related to PsyCap*

#### Mediating effects

The preceding discussion of theoretical relationships implicitly suggests that OVI and PsyCap is functioning as mediator among several of the variables comprised in this study. However, the following contains a discussion that explains and elaborates explicitly the rationale as well its hypothesis for the totally four mediators assumed to be identified in this study.

First, it is assumed that OVI mediates the relationship between PsyCap and CP. There are three interrelated underlying premises for proposing OVI as a mediator. First, a vision statement represent attempts to change employee behaviors [7]. In the existence of OVI, employees are consciously aware of undertaken changes based on what is prescribed and communicated by the vision of the organization. Second, motivation is a prerequisite for making changes. This represents the important role of PsyCap as a motivational driver for both OVI and CP. Third, OVI and CP share a common feature because they both focus on changes in a specific work role. On the other hand, CP and OVI differ in that OVI is about attention and intention to change (and thus attitude-like) while CP is about the actual manifestation (and thus behaviour-like) changes in how the work role is done. Taken together, when OVI increases because of an increase of PsyCap it should lead to an increase in the CP of employees. Consequently, OVI functions as the common denominator or nexus between PsyCap and CP. Therefore, driven by the PsyCap of employees, OVI becomes a central source to having a visionary mindset that in the next round could be reflected in employees CP in their respective work roles. While OVI is a source to visionary mindset, it also simultaneously functions as a boundary or a gatekeeper between PsyCap and CP. Specifically, OVI filters and decides what creative and innovative ideas who should pass further in such a way that it matches and are in harmony with the vision of the organization. Consequently, because of the central role that OVI seems to have, it is reasonable to assume it operates as a mediator in the relationship between PsyCap and CP. Consequently, the following hypothesis is proposed:

- **Hypothesis 6:** *OVI mediates the relationship between PsyCap and CP*

It is also expected that OVI of employees will mediate the relationship between employees perception of OA and CP. The rational for this is partially similar the same premises as discussed in the previous hypothesis. However, instead of having PsyCap as an initiator or originator to the domino-effect affecting OVI and CP it is now suggested that OA functions as this initiator, affecting OVI and CP. As mentioned in the previous discussion OA is an external oriented triggering factor (referring to employees perception of their organizational environment). Thus, OA just represents another type of initiating source compared to PsyCap, which were described as an internal oriented triggering factor (referred to as a personal-related condition or state). Consequently, when OVI increases because employees have a more favourable perception of OA, it should lead to an increase in the CP of employees. Therefore, as the common denominator or nexus in the relationship, OVI is both a source in having a visionary mindset while it simultaneously also functions as a boundary or an inner mental gatekeeper of vision between OA and CP. This latter aspect embraces how OVI selects what creative and innovative ideas are acceptable and could be passed further because it is in accordance and harmony with the vision of the organization. Consequently, because of its role it is expected that OVI will function as a mediator between OA and CP of employees. Consequently, based on this reasoning the following hypothesis is proposed:

- **Hypothesis 7:** *OVI mediates the relationship between OA and CP*

In the previous discussion it was proposed that PsyCap has a direct relationship with both OVI and CP. However, this study also proposes another alternative or additional route to how PsyCap can potentially relate to OVI and CP. Specifically, it is expected that PsyCap can act as a mediator. A mediator should explain the link between a predictor and a criterion variable. Specifically, it proposed that PsyCap play a mediating role between OA and the two variables OVI and CP. OA is, as also mentioned in the previous discussion, capable to develop, manage and change PsyCap employees in a positive direction. Therefore, the more employees perceive OA as positive the more it should drive or lead to an increase in the motivational muscle that PsyCap comprises. Furthermore, when PsyCap increases, stemming from employees having more favourable perception of OA, this should next lead to the higher level in both OVI and CP of employees in the organization. There are three reasons to expect PsyCap to act as a mediator. First, the idea finds support in previous research. For example, in a study of 103 service sales representatives, it was found that the PsyCap of employees functioned as a mediator between employees perception of organizational resources (referring to climate and leadership) and their performance outcomes such as innovative behaviour, employees sales performance and sales representatives general job engagement [23]. Second, support for PsyCap as a mediator also finds support in the Heskett et al.s service-profit chain model [36]. The basic idea and premises of the chain model are that internal factors of a service organization (e.g. OA) have an impact on how people think and feel (e.g. PsyCap) about their organizations, which next have an impact on their work role engagement and performance (e.g. OVI and CP). Third, further support for PsyCap as a mediator stems from the previously mentioned psychological-contract theory. Considering OA as part of employees psychological contract, it has impact or influence on what Lee et al. mentioned as employees efforts on behalf of the employer [34]. As such, it is reasonable to assume that PsyCap plays a mediating role both for employees vision implementation as well as their motivational effort explicitly manifested in their OVI and CP respectively. This reasoning leads to the two concluding hypotheses in this study:

- **Hypothesis 8:** *PsyCap mediates the relationship between OA and OVI*
- **Hypothesis 9:** *PsyCap mediates the relationship between OA and CP*

### Conceptual model

Figure1 provides a summary based on the discussion above. The conceptual model consists of three separate parts, organized in a causally related manner, and labelled *triggering factors*, *vision implementation* and *service effort*.

As seen in Fig. 1, OVI is reflected in the *vision implementation* among employees. *Service effort* is manifested by employees *creative performance* (CP). The *triggering factors* are represented by *psychological capital* (PsyCap) and *organizational attractiveness* (OA). PsyCap and OA represent two distinctive sources of triggering factors. OA is an external oriented triggering factor implying that it comes from outside the person and thus is an environmental-related factor. In contrast, PsyCap is labelled as an internal oriented triggering factor indicating that the source comes from within the person and consequently is a personal-related factor. Although OA and PsyCap are dissimilar, they have similarities because both are proposed to be initiators or generators of *vision implementation* (reflected in OVI) and *service effort* (reflected in CP).

As shown in Fig. 1, both OVI and PsyCap are suggested to be directly related to employees CP. Moreover, OA and PsyCap are proposed as direct triggering factors that promote employees OVI, and OA is proposed to be directly related to PsyCap.

In addition to the above mentioned direct effects, the relationships between OA, PsyCap and employees CP are both proposed to be mediated by OVI. Finally, the relationship between OA and the two variables OVI and CP is assumed to be mediated by employees PsyCap. Table1 summarizes all nine proposed hypotheses leading this study.

**Table 1:** Hypotheses leading this study

Hypothesized relationships	
H1	*OVI is positively related to CP*
H2	*PsyCap is positively related to OVI*
H3	*PsyCap is positively related to CP*
H4	*OA is positively related to OVI*
H5	*OA is positively related to PsyCap*
H6	*OVI mediates the relationship between PsyCap and CP*
H7	*OVI mediates the relationship between OA and CP*
H8	*PsyCap mediates the relationship between OA and OVI*
H9	*PsyCap mediates the relationship between OA and CP*

Note: *PsyCap* Psychological Capital, *OA* Organizational Attractiveness, *OVI* Organizational Vision Integration, *CP* Creative Performance

## Methods

This study has aimed to examine factors related to OVI, how OVI is adopted, absorbed or integrated among individual members of the organization, with a focus on the implementation of the vision among hospital employees. Consequently, as part of the health services research that focuses on individual-level innovations, we conducted a cross-sectional study in which Norwegian hospital employees (*N*=2000) were invited to participate. Participants in the study were all employed at hospitals situated in the inland counties of Norway. With over 10,000 employees, with a coverage of over 40 sites, the hospital organization is one of the largest health expert communities in its region. Initial contact was sought through the Director of Research (DOR), who disseminated all the information about the survey to division managers, staff unit and department managers. After several meetings and exchange of emails, the survey was developed to test the hypothesized relationships. Before sending out the survey to potential respondents, several pretests with two experts were performed to ensure the overall quality of the survey. As such, some redundant or ambiguous items were modified or deleted.

With the help of the DOR, an information email was sent to division managers and department managers to inform their employees of the study. Division managers and department managers were viewed as ambassadors to encourage and motivate employee participation in the survey. The survey information and URL were distributed by the DOR through emails to division managers and department managers, who furthered it to their employees. To maintain participant anonymity and avoid nonresponse bias, the study used a platform called *Nettskjema* (www.nettskjema.no). Irrespective of their degree of qualification, all hospital employees (*n*=2000) across 7 staff units and 10 divisions were encouraged to participate because the goal of this study was to examine generally the role of OVI in hospital organizations. Therefore, all specialized categories were summarized under a general category. For example, specialized nurse or senior nurse were summarized under the category Nurse, and specialized doctors or senior doctors were summarized under the category Doctor. Furthermore, we collected a total of *n*=1008 completed questionnaires, a response rate of 50.4%. As shown in Table2, personal characteristics were included in the survey. From Table 2, we can see that of the respondents in the study, 73% were female, reflecting the Norwegian context where the health sector is dominated by female workers [37]. About 37% of the hospital employees were under the age of 45years, 77% worked full time, and over 55% had been employed at the organization for more than 10years.

**Table 2:** Personal characteristics of the study sample (*N*=1008)

		%
Sex	Female	73.0
	Male	27.0
Staff role	Nurse	33.0
	Doctor	8.7
	Others (adm. Staff, other health professionals, etc.)	58.3
Employed	less than 5years	26.9
	between 6 and 10years	18.0
	between 11 and 20years	30.3
	more than 20years	24.8
Total work experience in public health	less than 5years	10.2
	between 6 and 10years	12.2
	between 11 and 20years	29.0
	between 21 and 30years	28.6
	more than 30years	20.0
Education	High school	15.7
	Bachelor degree	46.8
	Master degree	21.0
	Doctor of Philosophy degree	2.5
	Other	14.0
Part-time or full-time	part-time job	22.5
	full-time job	77.5
Age	younger than 45years	37.3
	between 46 and 55years	32.2
	older than 55years	30.5

### Instruments

This study covered four constructs: PsyCap, OA, OVI and CP. The claims used for the constructs in this study are listed in Table3. All claims used for the constructs are based on previous research. However, because none of the instruments have specifically been used in a Norwegian healthcare context before, there was a need to adapt claims to the study context, here Norwegian hospital organizations. The items used to capture the concept of PsyCap were adopted from Luthans et al. [38]. Items used to capture the concept of OA were adopted from Trybou et al. [29]. Items used to capture the concept of OVI were adopted from Liu [3] and Sltten and Mehmetoglu [39]. Finally, items used to capture CP were adopted from Zhou and George [40], Janssen [41] and Scott and Bruce [42]. It is important to note that in this study, the items used for the constructs PsyCap, OA, OVI and CP have all previously been validated in the healthcare setting [8, 43, 44]. However, the items were further adapted to fit the healthcare setting in the Norwegian context. A Likert scale from (1) strongly disagree to (7) strongly agree was used for all items. More importantly, the survey and all of its items used in this study are a part of a larger survey research project focusing on various aspects of employee-relations in hospital organizations. As such, the claims used in this study are appended accordingly (see Additionalfile1: Appendix 1).

**Table 3:** Constructs (Psychological Capital (PsyCap), Organizational Attractiveness (OA), Organizational Vision Integration (OVI), Creative Performance (CP)) and claims used in the study

Construct	Claims label	Claims
OA	OA1	(Hospital name) is attractive for me as a place for employment.
	OA2	I would recommend (Hospital name) as an employer for my friends.
OVI	OVI1	The management has informed me about the companys vision and aim.
	OVI2	I am familiar with the organizations vision and aim.
	OVI3	I am conscious of doing my job in line with the companys vision and aim.
PsyCap	PsyCap1	I feel confident that I can set goals for myself in my work area.
	PsyCap2	I am optimistic when it comes to my future at this organization.
	PsyCap3	When faced with challenges in my job, I can find alternative solutions to them.
	PsyCap4	I can find alternative ways to achieve my goals.
CP	CP1	I contribute creative ideas to solve challenges in my job.
	CP2	I contribute creative ideas to improve the quality of my job.
	CP3	I create new ideas to solve problems in my job.
	CP4	I search out new working methods or techniques to complete my work.
	CP5	I investigate and find ways to implement my ideas.
	CP6	I promote my ideas so others might use them in their work.
	CP7	I try out new ideas in my work.

### Data analysis

Based on the conceptual model, partial least-squares structural equation modelling (PLS-SEM) was used to test the hypotheses, using SmartPLS 3 software [45]. The first step in evaluating the PLS-SEM results involved examining a set of criteria for the reflective and formative measurement models. When the measurement models assessments were satisfactory, the next step was to assess the structural model. We followed the rules of thumb of Hair et al. [46, 47] to assess the quality of the measurement and structural model results. Based on the PLS-SEM results, mediating effects were also estimated and analysed using the bootstrapping test of Zhao et al. [48] and Nitzl et al. [49]. This bootstrapping test assesses whether the direct and indirect effects are statistically significant, and the combination of these two tests determines the degree of the mediation effect.

## Results

### Measurement model

OA and OVI were modelled as reflective constructs. To assess the reflective measurement models, we examined convergent validity, internal consistency reliability and discriminant validity. Convergent validity is the extent to which a claim correlates positively with alternative measures based on the same construct, and this was evaluated with loadings of the measures and average variance extracted (AVE). Internal consistency reliability is an estimate of the construct reliability based on the size of the correlations of the observed measures and was evaluated using composite reliability and Cronbachs alpha. Discriminant validity is the extent to which a construct is distinct from other constructs; in this study, as recommended by Hair and colleagues [46, 50], it is assessed using the heterotraitmonotrait (HTMT) ratio of correlation between constructs.

PsyCap and CP were modelled as formative constructs. Assessment of the formative measurement models were, as recommended by Hair et al. [46, 50], based on test of any multicollinearity for the indicators, the indicators weights and their significance as well as the indicators loading and their significance. All indicators had VIF values below 5, indicating no critical collinearity issues. Not all indicator weights, as is a measure of relative contribution to the formative constructs, were significant. Following Hair et al. [46], insignificant indicator weights should not be interpreted as poor measurement model quality, instead the focus should then be on the absolute contribution, represented as indicator loadings, and if these are significant, as is the case for our study.

As we can see from Table4, the evaluations of both the reflective and formative constructs support the view that this is a reliable and valid measurement model.

**Table 4:** Results of the measurement model for the reflective constructs of Organizational Attractiveness (OA) and Organizational Vision Integration (OVI) and the formative constructs Psychological Capital (PsyCap) and Creative Performance (CP)

*Reflective construct*	Claims label	Indicator reliability	AVE^a^	Composite reliability	Cronbachs alpha	HTMT criterion^a^
Rule of thumb		Loading >0.7	>0.5	0.70.95	0.70.95	HTMT interval does not include 1
OA	OA1	0.96	0.93	0.96	0.93	Yes
	OA2	0.96				
OVI	OVI1	0.88	0.81	0.93	0.88	Yes
	OVI2	0.93				
	OVI3	0.89				
*Formative construct*	Claims label	Loading	Loading sign.^b^	Weight	Weight sign.^b^	
PsyCap	PsyCap1	0.88	***	0.45	***	
	PsyCap2	0.85	***	0..41	***	
	PsyCap3	0.80	***	0.04		
	PsyCap4	0.84	***	0.27	***	
CP	CP1	0.73	***	0.05		
	CP2	0.76	***	0.25	***	
	CP3	0.86	***	0.33	***	
	CP4	0.81	***	0.04		
	CP5	0.83	***	0.10		
	CP6	0.89	***	0.34	***	
	CP7	0.82	***	0.10		

^a^*AVE* Average variance extracted, *HTMT* Heterotraitmonotrait ratio of correlations

^b^ *** *p*<0.01 is the significance level

### Structural model

**Fig. 2:**
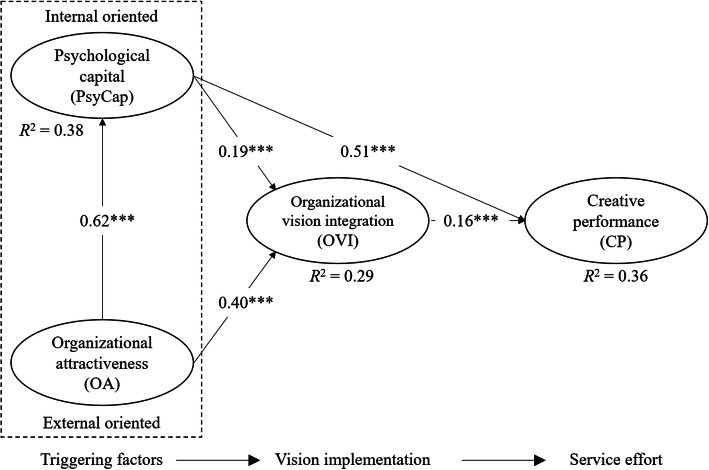
Results of the structural model of triggering factors and service effort of hospital employees vision implementation. Standardized coefficients (*** <0.01)

The direct effects in the structural model are shown in Fig.2.^1^ For the endogenous constructs, the models in-sample predictive power was examined using *R*^2^. Based on the rules of thumb [46, 50], the *R*^2^ values for PsyCap (0.38), OVI (0.29) and CP (0.36) were moderate. All the standardized direct-path coefficients were statistically significant at the 1% significance level. The path coefficient between OA and PsyCap was the highest at 0.62, the second-highest of 0.51 was between PsyCap and CP and the third-highest, 0.40, was between OA and OVI. The relationship between OVI and CP was positive (** = 0.16), supporting H1. H2 and H3 were also supported because the relationships between PsyCap and OVI and between PsyCap and CP were positive (** = 0.19 and ** = 0.51, respectively). Finally, there was a positive relationship between OA and OVI (** = 0.40) and between OA and PsyCap (** = 0.62), supporting H4 and H5.

As previously mentioned, when testing the mediation effect of the proposed hypothesis, as summarized in Table 1, we followed the approach of mediation analysis of a PLS-SEM, proposed by Nitzl et al. [49], as modified the mediation test in standard (covariance-based) SEM, introduced by Zhao et al. [48]. Simply stated, utilizing bootstrapping to assess whether the direct and indirect effects are statistically significant, we can determine whether there exist no-effect non-mediation, direct effects onlywithout mediation, complementary mediation, competitive mediation (direct and indirect effects are significant, but opposite direction) or indirect-only mediation. We tested two mediator effects of OVI and two mediator effects of PsyCap (Table5). OVI had a significant positive indirect effect (** = 0.032) and a complementary mediation effect on the relationship between PsyCap and CP, supporting H6. OVI indirect-only mediates the relationship between OA and CP (with an indirect positive effect of ** = 0.065), supporting H7. The positive indirect effect of PsyCap on the relationship between OA and OVI was significant (** = 0.118), implying a complementary mediation effect and support for H8. PsyCap showed a significant positive indirect effect (** = 0.313) and an indirect-only mediation effect between OA and CP, and thus H9 received support.

**Table 5:** Test of mediation effect of OVI and PsyCap

Hypo-thesis	Indirect effect	Mediator	Coeff-icient^a^	Bootstrapped bias corrected conf. Int.		Mediator effect^b^
H6	PsyCap CP	OVI	0.032^***^	0.017	0.053	Comp-lementary
H8	OAOVI	PsyCap	0.118^***^	0.069	0.165	Comp-lementary
H7	OACP	OVI	0.065^***^	0.040	0.097	Indirect-only^c^
H9	OACP	PsyCap	0.313^***^	0.259	0.354	Indirect-only^c^
^d^	OAPsyCap OVICP	PsyCap/OVI	0.019^***^	0.010	0.033	
^d^	Total indirect effect OACP		0.398^***^	0.351	0.435	

^a^ *** *p*<0.01 is the significance level

^b^ The PLS-SEM mediation analysis and classification of mediation effects are based on Zhao et al. [48] and Nitzl et al. [49]

^c^ The direct effect between OA and CP is not specified on our conceptual model. However, we estimated also a model included this direct effect, and found the direct effect OA CP not statistically significant (**=0.054, *p*=0.21). Based on that, in the mediation classification framework by Zhao et al. [48] and Nitzl et al. (2016), a significant indirect effect and insignificant direct effect imply indirect-only/full mediation

^d^ These two effects ((OA PsyCap OVI CP) and (Total indirect effect OA CP)) are not an direct part of our analysis, but included to report all possible effects in our conceptual model

## Discussion

This study aimed to examine factors linked to OVI. Consequently, the paper adds to the current research on organizational vision which is described as a key concept in the strategy and leadership literature [51]. Specifically, the study offers three main contributions. First, in contrast to the leadership perspective that has dominated previous research, this study examines the integration of organizational vision from an employee perspective. Second, it contributes to revealing the role that OVI plays for employees work performance, in this study manifested in their CP. As such it contributes to revealing whether OVI really matters and to what extent OVI can be described as a guiding force [15] to hospital employees work role activities. Third, it adds to our understanding of and insight into the direct impact of different types of triggering factors to OVI. Simultaneously, it contributes to revealing how different types of triggering factors indirectly (through OVI) are linked to employees work performance (represented by CP). To the authors knowledge, this is one of the pioneering studies to focus on OVI, as well as its antecedents (represented by OA and PsyCap) and effects (represented by CP), from a (hospital) employee perspective. Accordingly, this study offers a novel contribution to health services research.

Organizational vision is often referred to as the primary guiding force of all organizational activity [15]. Following this idea, OVI in this study is about whether hospital employees use the hospital vision as a guiding framework when making decisions and discretionary behaviors in their daily work roles [7]. The findings show that OVI has a direct impact on employees service effort manifested in their CP (** = 0.16). The impact of OVI, together with the direct impact of PsyCap (** = 0.51), explains almost 40% (*R*^2^ = 0.36) in employees service effort manifested in their CP. Consequently, hospital managers should be aware that OVI among hospital employees matters because it, together with PsyCap, constitutes a substantial power to guide CP of employees in hospital organizations.

Although few studies within health services research have examined the impact of OVI on CP in a hospital context, the results are supported within research undertaken in other types of service organizations where the human factor is important. One example of this is the study by Sltten and Mehmetoglu [18]. In their study, a total of 279 frontline employees in hospitality organizations participated. The findings revealed that employees level of strategic orientation (a similar concept to OVI) was positively associated with frontline employees work engagement and their innovative behaviour (a related concept to CP) as a part of their work role [18]. The value of organizational vision for employees performance is also supported in the study by Liu [3]. In their study, a total of 560 customer-contact employees from 50 branches (fast-food outlets) nationwide participated. The findings revealed that customer-contact positive perception of organizational vision was directly related to employees level of service effort in their respective work role. Similar to the concept of service effort in the study by Liu [3], service effort of CP in this study is manifested in the work performance of employees in their respective work role. However, CP in this study is an expanded type of service effort compared with the one in the study by Liu [3] and can best be described as an extra-role service effort. Because CP is not normally included in a formal work contract or as part of an in-role responsibility, CP goes beyond what is expected or should-do and thus it is about employees volunteering and their want-to-do extra effort reflected in their capability to think creatively and act innovatively (referring to CP). As found in this study, the OVI among employees is, together with their PsyCap, significantly related to employees extra-role service effort manifested in their CP.

The impact of OVI on CP also parallels ideas proposed in the study by Koryak et al. [51]. In their study, the authors examined, among several other factors, the impact of firms written vision on followers perception of their firms explorative activities. In their study, explorative activities referred to whether firms sought out new ideas, and were measured using items such as looking for novel technological ideas, exploring new technologies, creating products or services that are innovative. Thus, explorative activities in the study by Koryak et al. [51] match well what is embraced and constitutes core aspects of CP in this study. CP, which refers to thinking creatively and acting innovatively, is by its nature focused on performing explorative activities. Similar to our study, Koryak et al. [51] assumed that organizational vision should motivate, guide attention and action and lead to more explorative activities following daily operations. Although these reasons are both plausible and logical from a theoretical point of view, the authors did not receive empirical support in their study results. Consequently, the findings in our study differ from Koryak et al. [51] because we found a significant relationship between OVI and CP. However, some potential reasons could explain why Koryak et al. [51], in contrast to our study, did not receive empirical support. Specifically, we suspect two plausible reasons could potentially explain the insignificant findings in Koryak et al. Both reasons are based on how organizational vision is focused on in Koryak et al., which constitute a major contrast to this study. First, in the study by Koryak et al., a majority of participants (85%) were top executives (CEO). Consequently, the authors of the study took a leadership perspective and not an employee perspective as done in this study. Second, the concept of organizational vision in Koryak et al. [51] focused solely on the communicative aspect of organizational vision. Participants (referring to leaders) were asked two questions about firms vision, namely, (i) does your company have a written vision? and (ii) have you talked to your employees about your vision for the company in the last 6months? Consequently, the focus on organizational vision in Koryak et al. [51] is on the senders (referring to leaders) and not on the receivers (referring to employees) of organizational vision which is done in this study. Although it is important to communicate organizational vision well throughout the organization, it is fundamental and critical that organizational members themself perceive if they are informed, familiar with and conscious about doing their job in line with the companys vision and aim. This latter aspect is what OVI is about in this current study. Based on this reasoning, one way to interpret or understand the insignificant findings between organizational vision and explorative activities in Koryak et al. [51] is to say the findings (just) confirm that it is not satisfactory enough for leaders to just communicate the vision throughout the organization. Specifically, it confirms that communication of organizational vision is not the same as achieving OVI among employees. Consequently, the relationship between OVI and CP revealed in this study is an implication for hospital leaders to have a clear focus on how well OVI is understood among organizational members. Thus, an explicit formula to potentially achieve success for hospital leaders, regarding organizational vision, can be pronounced by living (or leading) the following slogan: *Communicate to integrate*.

The results in this study further reveal that when OVI is clearly present in hospital employees minds and feet, it can have an impact on hospital employees CP manifested in their respective work role. Furthermore, it is also essential for hospital managers to understand those factors that positively foster employees OVI as well as employees CP. In this study, two types of triggering factors were proposed to be related to OVI and CP. PsyCap is proposed to be an internal or a personal-oriented triggering factor while OA represents an external or an environmental-oriented triggering factor. As explained in the previous discussion, the triggering factors differ in their orientation. However, the findings reveal that PsyCap and OA are interrelated, and in different ways, have an impact on both employees OVI and their CP.

PsyCap was found to have a direct impact on employees level of OVI (** = 0.19) as well as having a direct impact on CP (** = 0.51). Furthermore, based on the mediator test suggested in Zhao et al. [48], it was found that OVI operates as what Zhao et al. term complementary mediation [48]. A complementary mediation signifies that two pathways lead to CP. The first works directly from PsyCap to CP and the second means that OVI has a mediating effect between PsyCap and CP. It is important to recognize that these two routes do not substitute each other but co-exist and act as complementary impact. PsyCap is about a persons positive psychological state of the individual towards positive development [20]. Consequently, based on the findings in this study, those resources embraced in employees PsyCap are important triggering factors and motivators to both employees OVI and their CP. Previous research supports that PsyCap (or parts of PsyCap) is linked to aspects of employees CP [23,25]. However, to the authors knowledge, this is among the first studies in health services research to reveal the role PsyCap plays, both directly and indirectly, on employees OVI as well as employees CP. In the literature, PsyCap is described as a resource a person possesses. Furthermore, this resource embraced in a persons PsyCap is characterized as being relatively controllable, which implies that PsyCap is open to development [21] and manageable for effective work performance [21]. An implication of this is the importance of hospital leaders continuously to cultivate and develop PsyCap among their hospital employees and specifically in a positive way manage those four resources that PsyCap consists of (referring to (i) hope, (ii) efficacy, (iii) resilience and (iv) optimism).

Although leaders can help develop those resources of PsyCap, the results of this study further reveal other types of triggering factors that are related to employees PsyCap. Specifically, OA was found to have a direct impact on PsyCap (** = 0.62) and explains 38% of the variance in PsyCap. OA, as an external oriented triggering factor, is about how employees attitude [28] towards the environment in the organization in which they are employed is considered as good or bad. OA has substantial influence or managing impact on PsyCap. Although OA is highly central for employees PsyCap, the findings reveal that OA influences OVI and CP. When considering OVI, which is the most focused concept in this study, the findings reveal that OA has a direct impact on OVI (** = 0.40). It is important to note that the impact of OA (an external environmental-oriented trigger), is twice the size of the direct effect of PsyCap (an internal personal-oriented trigger) on OVI (** = 0.19). It reveals that OA is the primary triggering source for OVI among employees. Consequently, hospital leaders should be aware of the important role OA plays for the successful achievement of integrating the organizational vision among organizational members.

However, although OA have a dominating direct impact on OVI, OA together with PsyCap explain almost 30% (*R*^2^ = 0.29) of the variance in OVI. Furthermore, the analysis shows that OA has another pattern that relates to OVI. Based on the mediation results presented in Table 5, this study found that OA also functions as what Zhao et al. term complementary mediation [48]. Specifically, this means that in addition to having a direct impact on OVI, the impacting power of OA on OVI simultaneously works through the resources embraced in employees PsyCap.

Finally, OA also plays a role when it comes to employees CP. However, based on the mediation test of Zhao et al. [48], the findings reveal that OA does not act as a complementary mediation but in contrast acts as an indirect-only mediation. In total, two indirect-only mediations between OA and CP were found. The first indirect-only mediation works through OVI and the second indirect-only mediation works through PsyCap. When comparing the different patterns of relationship, it reveals that OA plays a multifaceted role and is highly central for OVI among employees. In addition, the mediation results in Table 5 reveal that OA has an indirect effect on OVI as well as on employees PsyCap and employees CP. Although limited research within health services research has examined the multiple impacts of OA, as done in this study, the findings highlight the importance of hospital leaders to focus on OA. As Trybou et al. noted: hospital attractiveness is of major importance [29]. Consequently, hospital leaders should follow the recommendation by Sltten et al. who advocate that OA is something that needs to be focused on, maintained, and cultivated [28].

### Limitations and future research

According to Kantabutra and Avery more research is still needed into characteristics of power visions and vison realization factors [4]. This study has contributed to revealing that organizational vision really matters when considered from an employee perspective. However, research on this issue within health services research is relatively absent and can be described more or less as a black box. There is a need for substantial research on a range of aspects within the domain of organizational vision. Based on this study, three areas to be focused on in future research are suggested.

First, and this is most fundamental, considering the focus on OVI in this study. Although the way OVI is defined in this study functioned well, there is a need for more research to capture the true nature of the aspect integration in the concept of OVI. Integration is a relatively complex phenomenon. Integration can be described as the act of bringing together smaller components into a single system that functions as one. Based on this, future research should try to reveal whether the concept of OVI consists of one or several subdimensions. Should OVI be considered as a formative or reflective concept? Taking an employee perspective, future research on OVI should focus on the act in time regarding integration. Specifically, research should focus on the process of how OVI is manifested among employees in an organization. What does a positive OVI process normally look like? Why is it that some employees do not integrate an organizational vision? Future research could also look at integration by considering how employees perceive aspects related to the explicit formulation and aspiration communicated through the formal written vision of organizations. What are the ingredients of a formal written vision that are capable of capturing the minds and feet of employees in a hospital organization? How does an organizational vision motivate and engage hospital employees? Is there a difference in OVI when employees contribute in the process to develop a new vision, compared with when they are just presented a new vision? Because of the complexity of OVI, it is highly recommended to do more qualitative research on OVI. Moreover, such qualitative studies could next be followed up and tested on a large scale, using quantitative methods, to reveal the generalizability of findings both within and across contexts.

Second, this study contributed to revealing two types of triggering factors that had an impact on OVI. Both PsyCap, as an internal (personal) oriented triggering factor, and OA, as an external (environmental) oriented triggering factor, have an impact on OVI. As previously mentioned, two types of triggering factors explained about 30% (*R*^2^ = 0.29) of the variance in OVI. Consequently, there is considerable variance left in OVI to be explained. Based on this explanation gap in OVI, future research should include other types of both internal and external triggering factors. Considering external oriented triggering factors, future research could examine how different types of organizational culture act as a triggering factor for OVI. For example, one could study the impact of the four culture types proposed by Cameron and Quinn [52]. The four mentioned in their framework are (i) clan culture, (ii) adhocracy culture, (iii) market culture and (iv) hierarchy culture. Other potential types of organizational culture would be to examine the impact of innovative culture [53] or internal market-oriented culture [28, 54] on OVI. Other dimensions of external oriented triggering factors to OVI would be to focus on how different leadership styles can foster OVI. In the literature, there is a range of possibilities of leadership styles to include such as leadership autonomy support, empowering leadership, authentic leadership, ambidextrous leadership or several other leadership styles mentioned in the literature. When considering internally oriented triggering factors, future research could examine how variation in employees inner attachment to the organization has an impact on OVI. One such factor would be to study the impact of what the literature labels as employees affective commitment. Affective commitment is defined as employees positive emotional attachment to the organization [55]. Another internal oriented triggering factor is the relatively new concept of thriving [56] which is defined as the psychological state in which individuals experience both a sense of vitality and learning at work [57]. These external and internal oriented triggering factors are only a few examples, among several other potential triggering factors, that could be included as impacting factors to OVI in future research.

Third, this study found that OVI, based on the mediation test of Zhao et al. [48], acts as both complementary mediation and indirect-only mediation in relation to CP. It is highly recommended that future research uses sophisticated statistical tests to reveal types of mediating effects. Such statistical tests bring more nuanced knowledge and insight about both the role of OVI as well as identifying potential patterns on how OVI is related to service effort such as the level of CP among employees in organizations. Future research could also relate OVI to other types of service effort relevant to health care organizations such as the level of service quality, the level of productivity in the delivery of services or others. Consequently, based on the specific recommendations in each of the three areas regarding future research related to OVI, it is well reflected and is summarized in the following statement by Foster and Akdere: There is much room for more research and investigation to be completed in the area of organizational vision [58].

## Conclusions

Previous research has predominantly taken a leadership perspective when studying organizational vision. The contribution of this study is to examine factors related to OVI from an employee perspective using hospital organizations as the empirical setting. The findings reveal that to achieve OVI successfully among hospital employees, hospital leaders need to be conscious of the complex configuration of influences of both personal as well as environmental-related factors.

## Supplementary Information

**Additional file 1: Appendix 1.** Questionnaire developed for this study.

## Data Availability

The datasets used and/or analysed during the current study are available from the corresponding author on reasonable request.

## Abbreviations

PsyCap: Psychological capital
OA: Organizational attractiveness
OVI: Organizational vision integration
CP: Creative performance
PLS-SEM: Partial least-squares structural equation modelling
HTMT: Heterotraitmonotrait
AVE: Average variance extracted
DOR: Director of research
NSD: Norwegian Centre for Research Data

## Footnotes

1 Before the structural model was assessed, multicollinearity between the latent constructs was examined by looking at the variance inflation factor (VIF) values, where VIF values above 5 indicate multicollinearity issues [50]. All VIF values were lower than 2, indicating no multicollinearity problems.
